# Supplementary material for: Critical scaling of novelty in the cortex
Source: Nat Commun. 2026 Jan 10;17:1555. doi: 10.1038/s41467-025-68277-0 (PMC12894744; doi:10.1038/s41467-025-68277-0)
Supplement: Supplementary file 1 — Supplementary Information [file 41467_2025_68277_MOESM1_ESM.pdf]

Supplementary Information

Critical Scaling of Novelty in the Cortex

Tiago L. Ribeiro, Ali Vakili, Bridgette Gifford, Raiyyan Siddiqui, Vincent Sinfuego, Sinisa Pajevic, and Dietmar Plenz[†]

Section on Critical Brain Dynamics, National Institute of Mental Health, Bethesda, MD, USA

[†] Correspondence: plenzd@mail.nih.gov

Supplementary Figures

Supplementary Figure 1

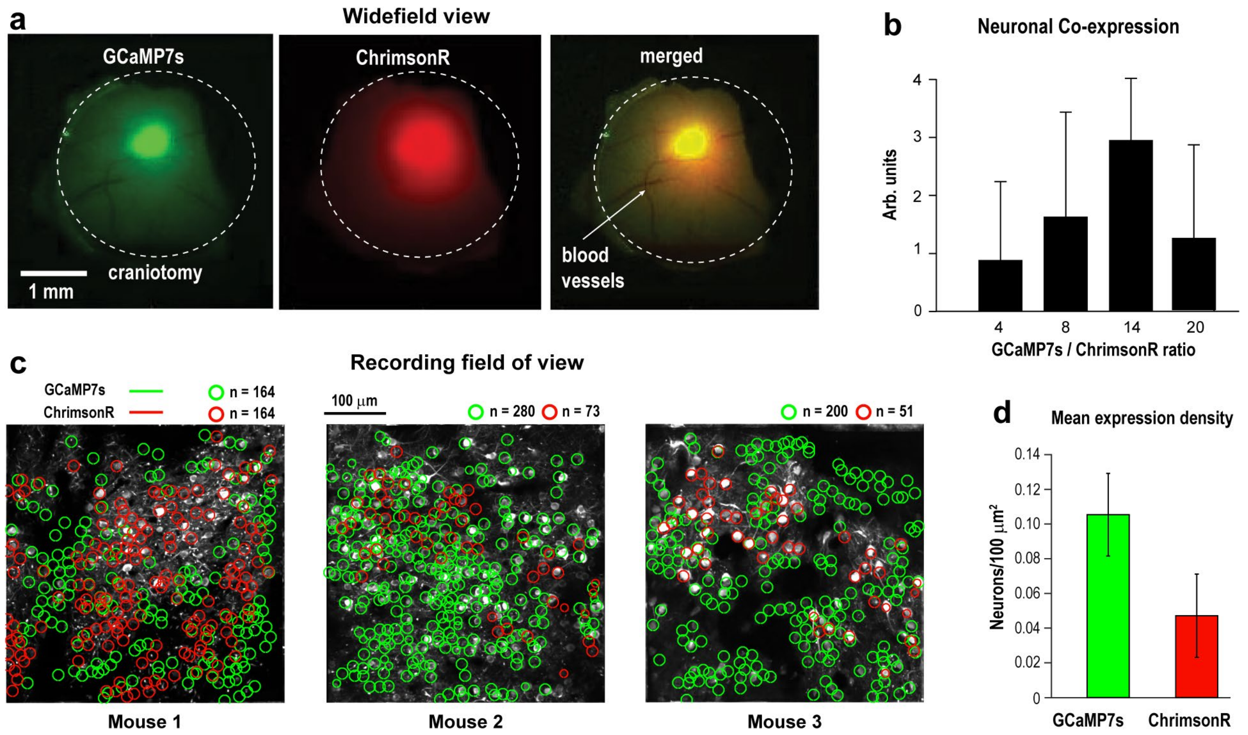

Supplementary Fig. 1 | Co-expression of Gcamp7s & ChrimsonR in the visual cortex of mice. **a** Widefield image showing GCaMP7s (*green*) and ChrimsonR (*red*) expression, with the merged image indicating co-expression (single mouse). **b** Optimal co-expression achieved with a virus titer ratio of 14:1 (mean ± SD, *n* = 23 mice). **c** Examples of individual neurons with significant GCaMP7s (*green*) and ChrimsonR (*red*) expression from 3 mice. **d** Mean ± SD (*n* = 3) expression density showing twice as many neurons positive for GCaMP7s compared to ChrimsonR (from c).

Supplementary Figure 2

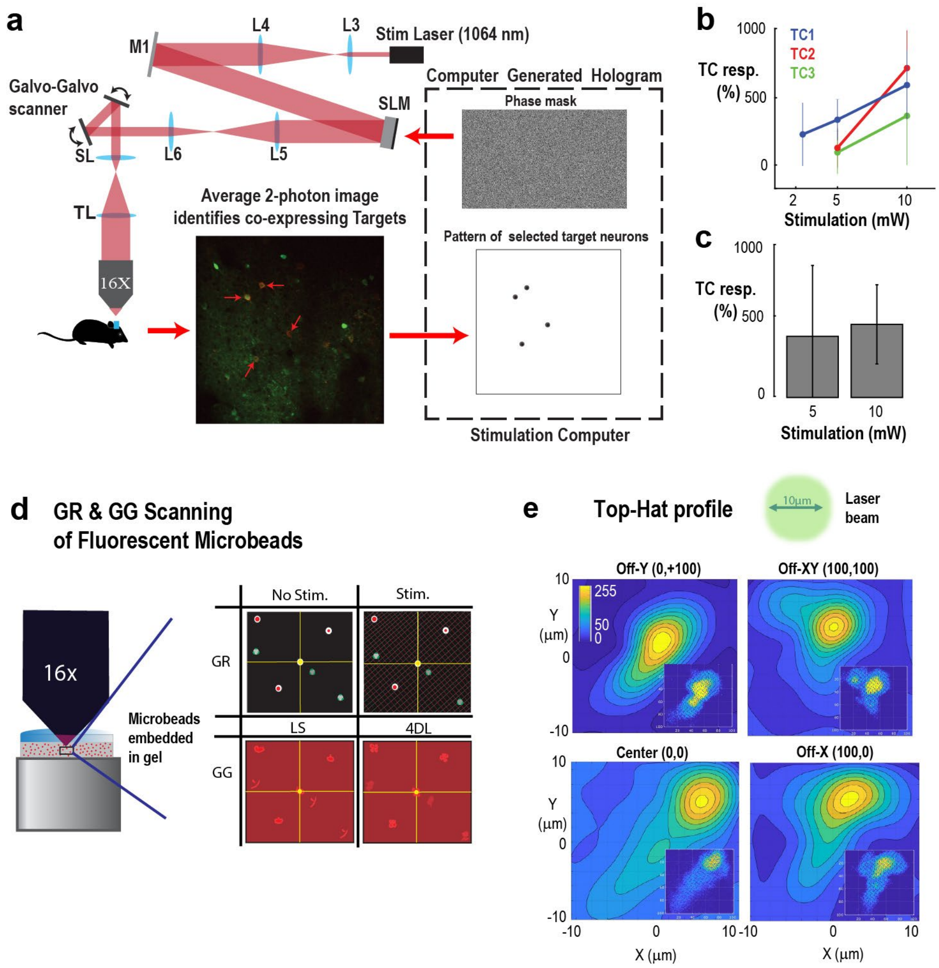

Supplementary Fig. 2 | Schematic design and evaluation of the stimulation path using spatial light modulation. a Laser light (Light Conversion, 1064 nm, 600 kHz, <100 fs) is expanded to fill the area of the spatial light modulator (SLM; Meadowlark, 1920×1152 resolution). The hologram is resized to fit the Galvo-Galvo scanners (~3 mm diameter), which guide the stimulation beam in the focal image plane (Nikon 16X, 3 mm working distance, 12.5 mm effective focal length). Target cells (TC) expressing both GCaMP7s and ChrimsonR (opsin with tdTomato tag) are selected from the mean 2PI image of the field of view from both red and green channels for stimulation and simultaneous estimation of the evoked spike count. Map of TC informs a hologram of top-hat patterns with diameter of 10 μm for the SLM. Optical power was adjusted to 5—10 mW per TC, depending on the depth and expression level. **b** Spiking above base line increases with stimulation power (*n* = 3 TC; 100 ms stimulation duration). **c** No difference in evoked spike count for 5 and 10 mW stimulation across all TC (*n* = 15 TC). **d** Schematics of the stimulation xy-profile reconstruction using fluorescence microbeads. Galvo-Galvo (GG) controlled stimulation of gel-embedded microbeads (3 μm diameter) and simultaneous Galvo-Resonance (GR) 2PI of elicited response. *Circles*: Individual microbeads before and during stimulation. **e** Experimentally reconstructed and smoothed xy-profile of a top-hat stimulation (10 μm diameter; 5 mW) at 4 different positions within the field of view at center (0,0), off-x (+100 μm, 0), off-y (0, +100 μm), and off-xy (+100 μm, +100 μm). *Inset*: Non-smoothed reconstruction. *Error bars* (b, c): mean ± SD.

Supplementary Figure 3

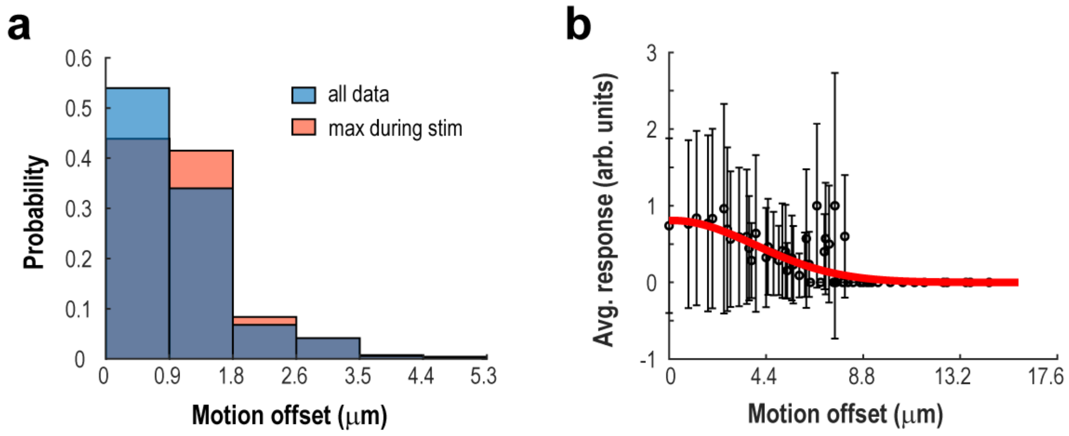

Supplementary Fig. 3 | Analysis of motion correction from processed recordings reveals low amount of motion artifact affecting stimulation. a Distribution of motion offsets observed for each frame over all recordings. *Blue*: all data (n = 892k). *Orange*: maximum displacement observed during stimulation for each trial (n = 9316). **b** Spatial profile of stimulation response from targets measured using motion offset in trials during stimulation. Note that responses drop to approximately zero in frames with an offset above ~4.4 μm. Mean ± SD.

Supplementary Figure 4

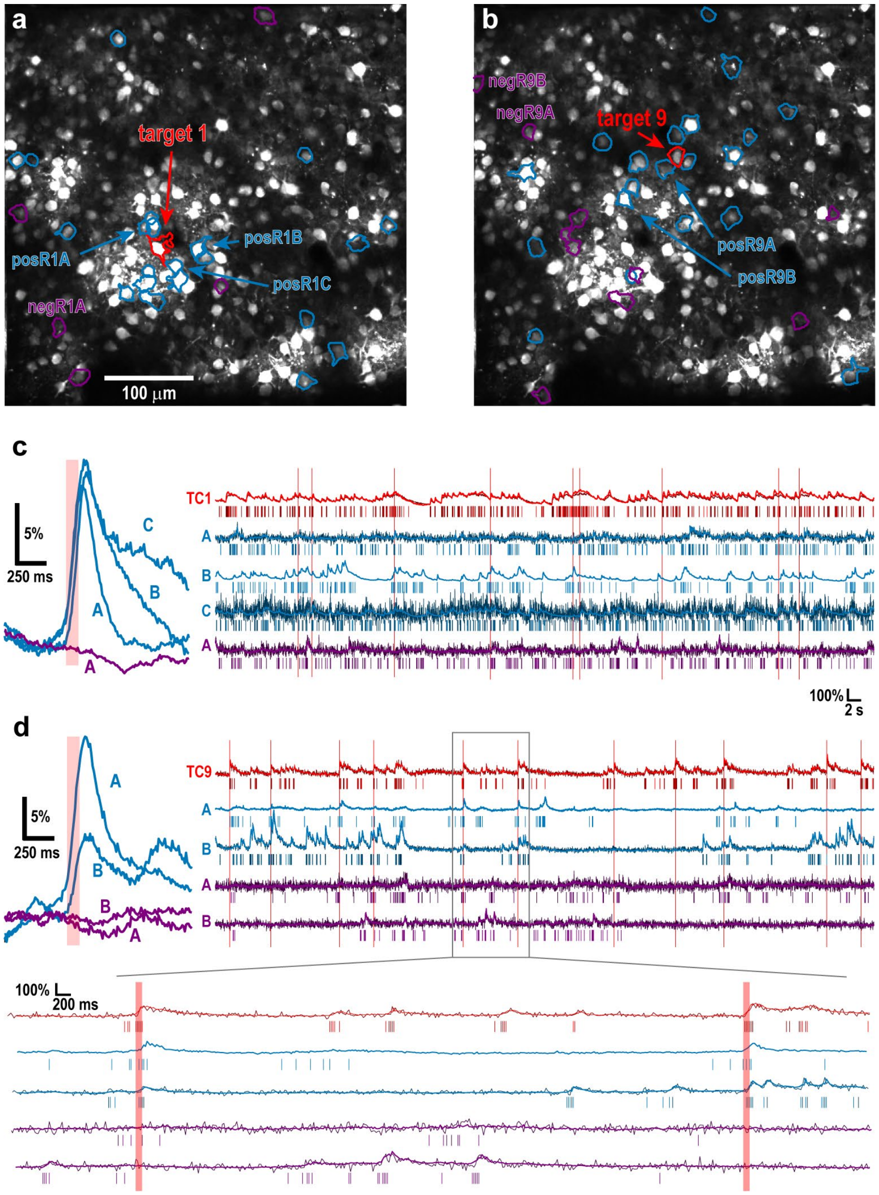

Supplementary Fig. 4 | Example recording with activity from two example targets and some of their significant responders highlighted. a Example field of view with target 1 highlighted in *red* and its positive (*blue*) and negative (*purple*) responders indicated. **b** Same recording as in a but now highlighting target 9 and its significant responders. **c** *Left*: time average fluorescent response to stimulation for the significant responders labeled in a. *Right*: example raw (*dark*) and denoised (*light*) fluorescent traces (*lines*) and deconvolved spikes (*bars*) for the labeled neurons in a. **d** *Left*: time average fluorescent response to stimulation for the significant responders labeled in b. *Right*: example raw (*dark*) and denoised (*light*) fluorescent traces (*lines*) and deconvolved spikes (*bars*) for the labeled neurons in b. *Bottom*: zoom in from the right panel above.

Supplementary Figure 5

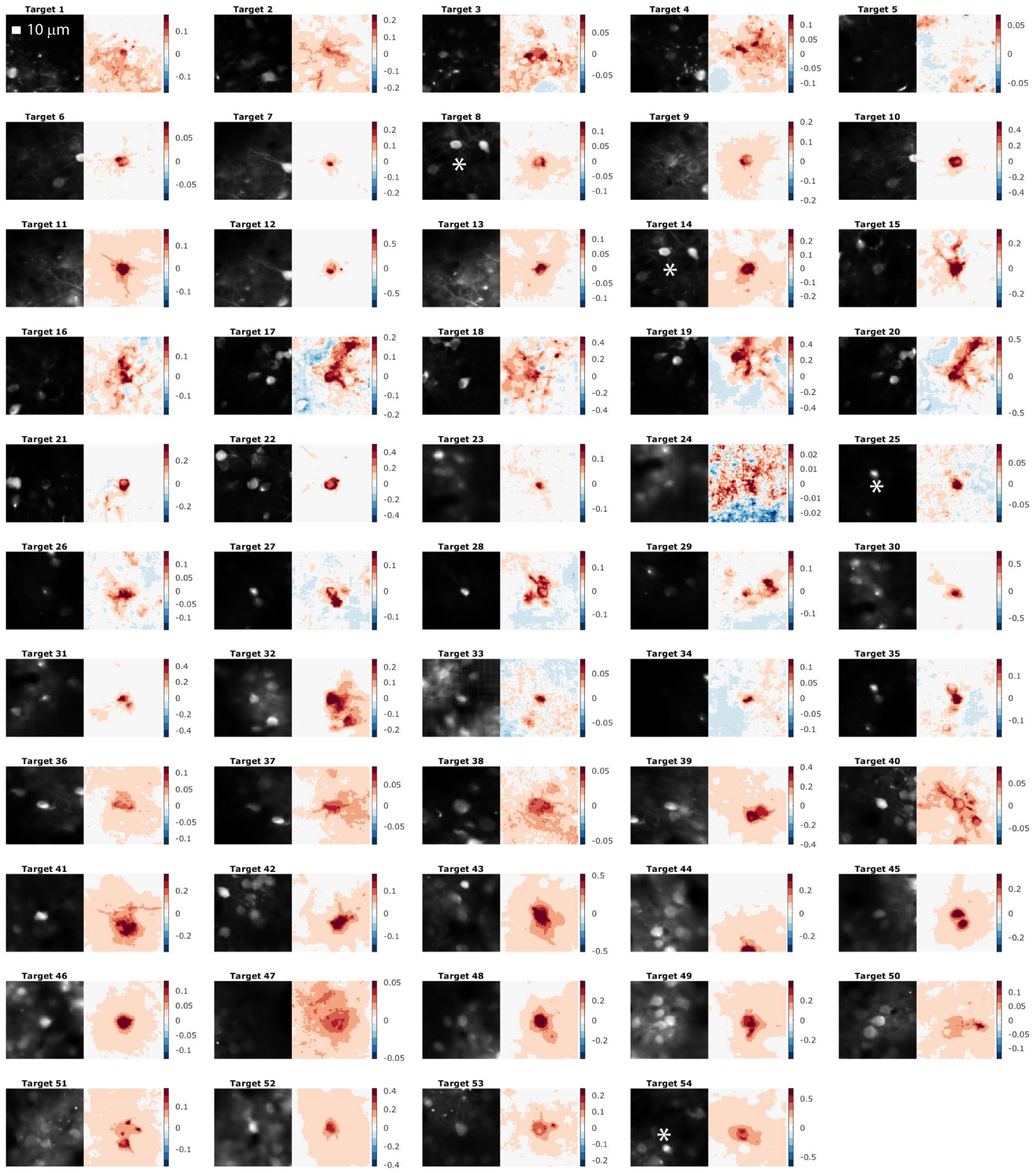

Supplementary Fig. 5 | Summary of isolating individual pyramidal neurons in layer 2/3 of the primary visual cortex during rest using holographic stimulation and 2PI. For each target cell (TC), mean luminance after motion correction and denoising (*left*) and the ΔF/F in response to stimulation in a 100×100-pixel area (~88×88 μm) centered on the TC (*right*) are shown. *Asterisks* are visual guides for TC position.

Supplementary Figure 6

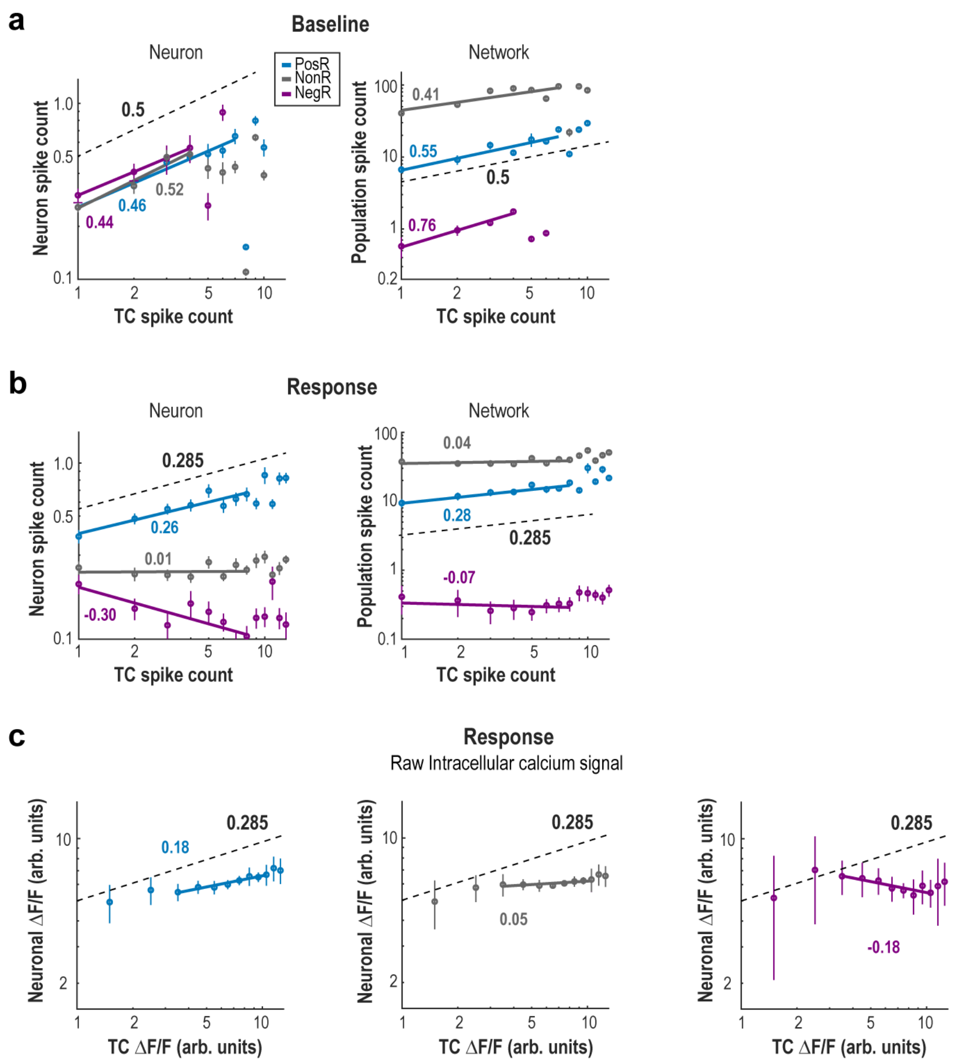

Supplementary Fig. 6 | Spike count in all subpopulations scale with spike count of target cells during baseline activity, but only Positive Responders scale during stimulation. a Mean spike count on PosR (*blue*), NonR (*grey*) and NegR (*purple*) as function of spike count of TC shown in log-log plot for baseline activity. Power law fits are indicated by solid lines, with the obtained exponents displayed. *Left*: mean calculated per neuron. *Right*: mean calculated after summing over the population. **b** Same as in a, but during holographic stimulation. Note the positive scaling for PosR but not NonR and NegR. **c** Response scaling calculated using the fluorescent traces (ΔF/F) exhibits positive scaling only in PosR. Summary over all mice and experiments. *Error bars*: mean ± SE (a, b) or SD (c).

Supplementary Figure 7

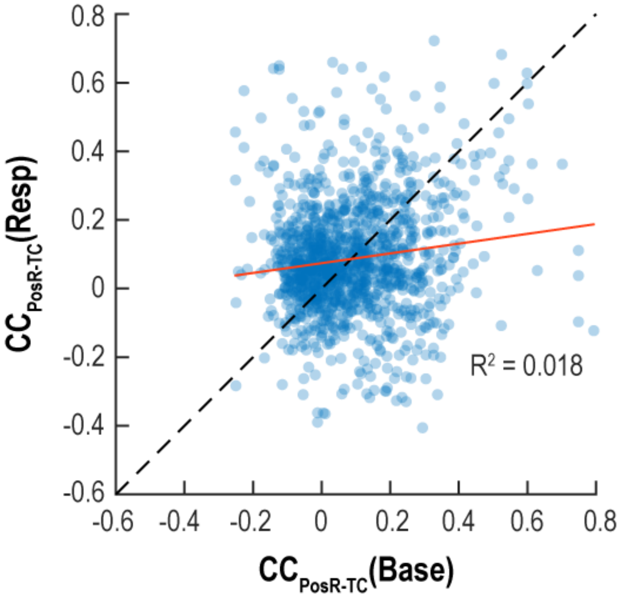

Supplementary Fig. 7 | Weak change in correlations between PosR and TC during stimulation in comparison to baseline. For each PosR-TC pair, the correlation of their spike count during stimulation is plotted against the baseline correlation. There is a small shift towards higher values during the response phase compared to the baseline (see Fig. 2c), but the overall correlation between the two measures remains weak. This indicates that the stimulation of TCs activates both existing and new sub-networks ($R^2 = 0.018$; linear regression; $p < 10^{-5}$, based on the t-statistic of the two-sided hypothesis test).

Supplementary Figure 8

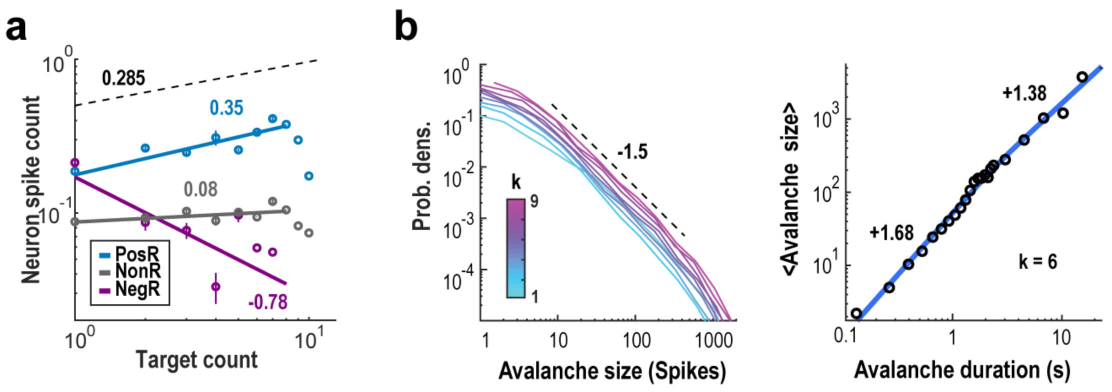

Supplementary Fig. 8 | Response scaling and avalanche analysis remain qualitatively unchanged even without denoising from Deep Interpolation. a Average response from PosR (*blue*), NonR (*grey*) and NegR (*purple*) as function of TC spike count during stimulation is shown for datasets processed without denoising (i.e. without Deep Interpolation). *Error bars*: mean ± SE. **b** Avalanche analysis for datasets processed without denoising. *Left*: Distribution of avalanche sizes for different coarse graining levels *k* (*color bar*). *Right*: Mean avalanche size as function of avalanche duration at *k* = 6 showing an exponent below 2 for the short avalanches regime, in line with previous results obtained from noisy datasets (Ribeiro, Capek et al., 2023).

Supplementary Figure 9

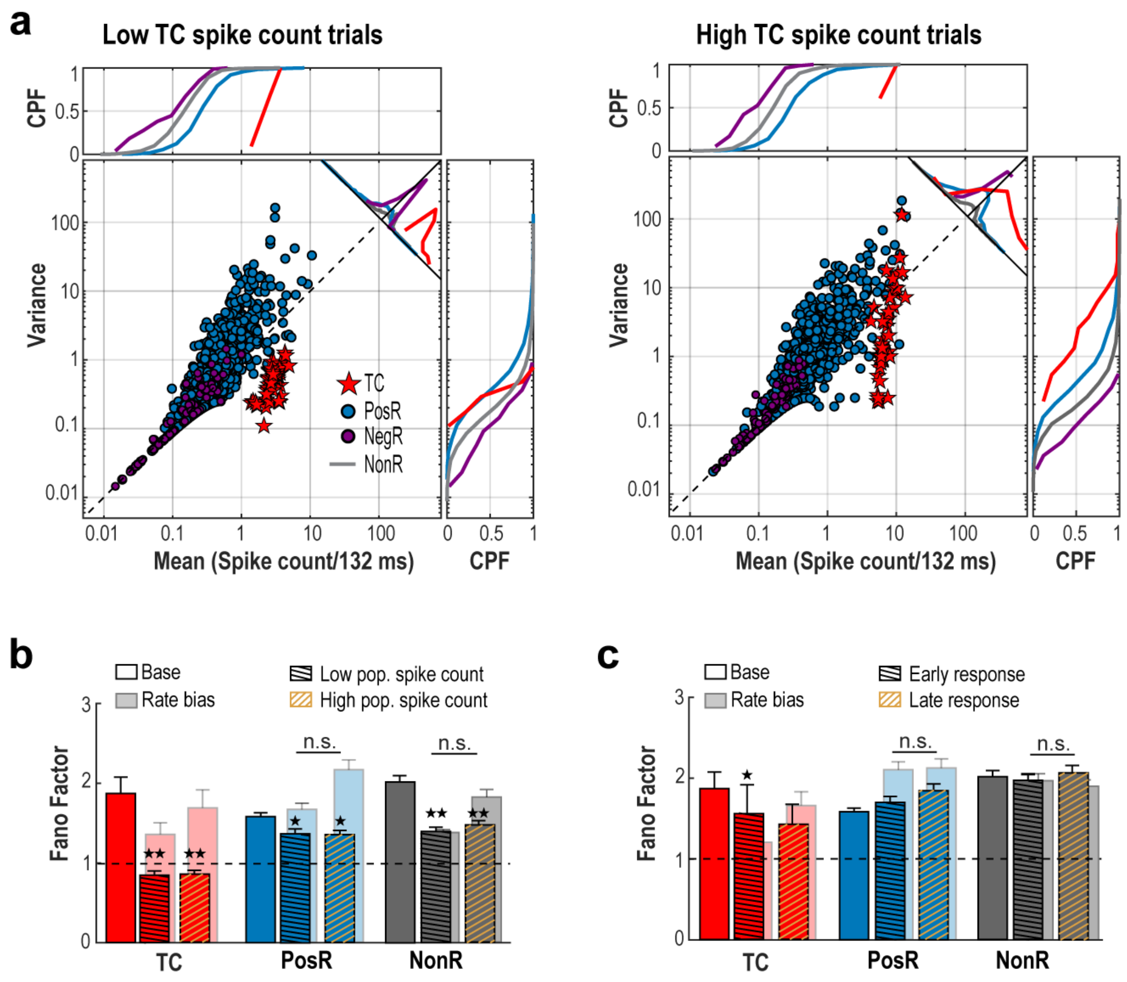

Supplementary Fig. 9 | High response variability in PosR and NonR does not originate from variability in evoked spikes of TC. a Summary statistics of mean TC trials separated by low and high target counts. Note the remaining high variance of PosR despite the large drop in TC variance. No change in Fano Factor (FF) when subdividing into TC trials with high or low preceding population spike count (**b**) or early (first half of the response window) vs. late (second half of the response window) spiking responses (**c**). *Error bars* (b, c): mean ± SE across experiments, with n = 54, except for: (b) TC Low/High pop n = 17/16, PosR Low/High pop n = 49/49; (c) TC Early/Late n = 33/13, PosR Early/Late n = 53/53. When comparing FF (b, c), 1 & 2 stars indicate $p < 0.05$ & 10^{-4}, respectively, using one-tailed Wilcoxon rank sum test (b: TC Low pop $p = 8.5\times10^{-8}$, TC High pop $p = 2.4\times10^{-7}$, PosR Low pop $p = 0.008$, PosR High pop $p = 0.003$, NonR Low pop $p = 3.0\times10^{-8}$, NonR High pop $p = 9.6\times10^{-7}$; c: TC Early $p = 2.9\times10^{-4}$). All comparisons are done against the baseline of the respective neuronal group.

Supplementary Figure 10

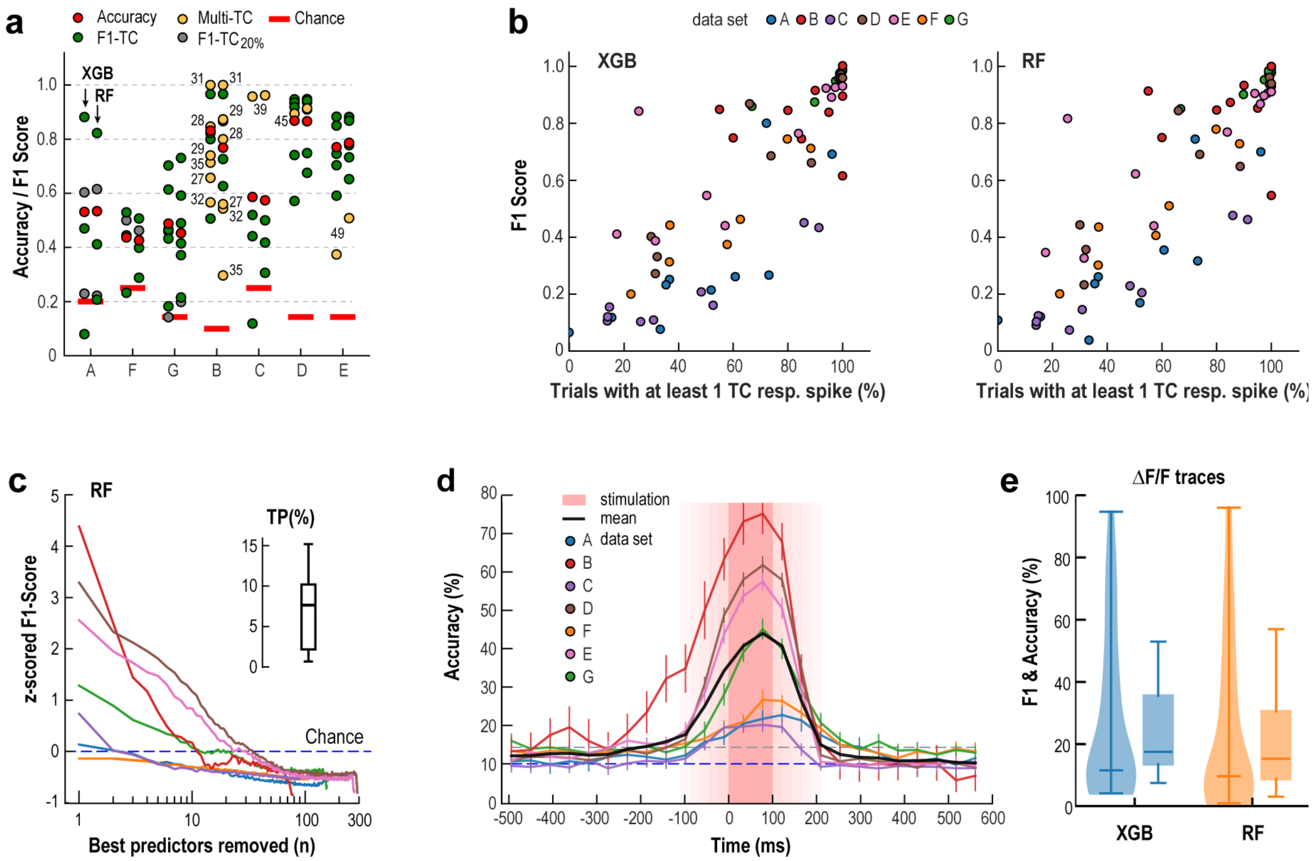

Supplementary Fig. 10 | Decoding accuracy, stimulation efficacy and impact of deep interpolation. a Overview of F1-scores for TCs (*green*) and TCs that did not reach 20% of spike success (*gray*) upon stimulation. F1-scores are compared to chance level (*red bars*) and overall accuracy (*red circles*). XGBoost (XGB; *left*) and Random Forest (RF; *right*) results are very similar across all datasets. Potential multi-cell targets (cp. Supplementary Fig. 5; TCs 27-29, 31-32, 35, 39, 45 and 49) are labeled and marked in *yellow*, and overall do not show above-average decoding success. **b** For each data set, stimulation success (at least 1 spike) varied among TC. Target stimulation success positively correlated with decoding power, i.e. F1-score, of a TC in XGB (*left*) and RF (*right*). **c** Z-scored F1-scores calculated with the Random Forest method for each TC and then averaged, for each dataset (same color code as in Fig. 4), as function of number of best predictors dropped (cp. XGB in Fig. 4c, *right*). *Inset*: Average number of best predictors dropped before F1-scores reach chance levels. **d** Deep Interpolation slightly advances stimulus information and significantly increases accuracy thereby slightly prolonging transient response time. Analysis done over 132-ms window (cp. Fig. 4f, 22-ms window). The enhanced accuracy achieved with Deep Interpolation demonstrates the method's noise-reducing benefits, which improve the model's ability to capture relevant neural signals. However, it also shows that it undermines our ability to study temporal progression of activations, as its window of smoothing ranges across 30 frames. *Error bars*: mean ± SD. **e** Low (but still above chance) F1 and accuracy when XGB and RF are trained on the raw ΔF/F traces. Accuracy calculated over n = 140 samples, *F1* scores calculated over n = 880 samples. *Box/violin plots* (c, e): *whiskers* represent min, median & max, while *box edges* represent 25th and 75th quartiles.

Supplementary Figure 11

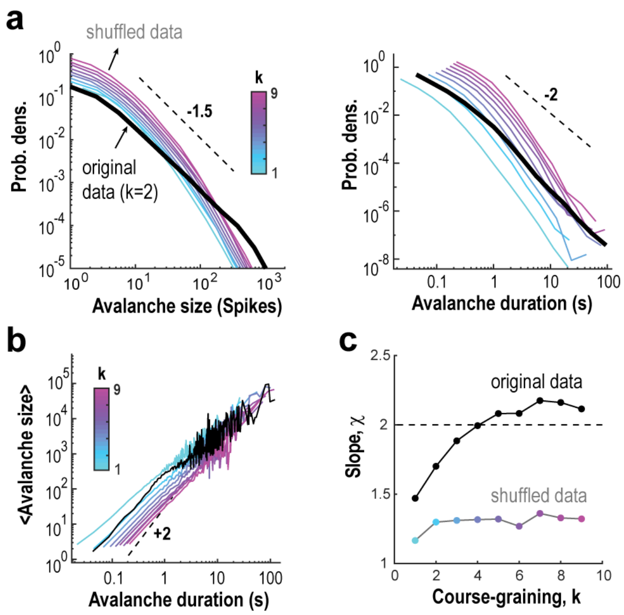

Supplementary Fig. 11 | Avalanche analysis on shuffled data results in deviation from the scale-free properties and scaling observed for original data. a Avalanche analysis for circular-shifted datasets. Distribution of avalanche sizes (*left*) and duration (*right*) for different coarse graining levels *k* (*color bar*). The same for original data at k = 2 is also added for reference (*black*). **b** Mean avalanche size as function of avalanche duration for different coarse graining levels (*color bar*). The same for original data at k = 2 is also added for reference (*black*). **c** Scaling exponents measured from original (*black*) and shuffled (*gray/colors*) data show contrasting results, with only the original data being able to achieve a parabolic scaling (exponent 2) with coarse graining.

Supplementary Figure 12

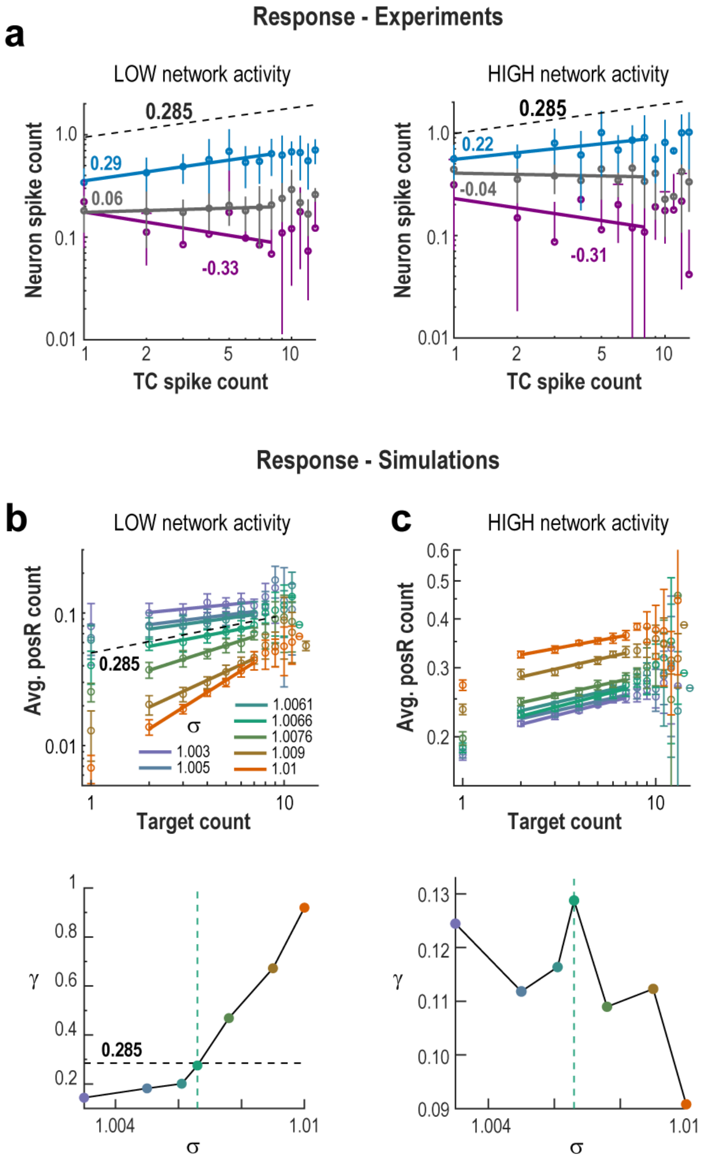

Supplementary Fig. 12 | Spike count in subpopulations scale similar with spike count of target cells during low and high avalanche activity states. a Mean spike count on PosR (*blue*), NonR (*grey*) and NegR (*purple*) as function of spike count of TC, in log-log, during holographic stimulation. Power law fits are indicated by solid lines, with obtained exponents shown. Separating trials with preceding low (*left*) or high (*right*) baseline network activity. Experimental data. **b** Mean response of PosR as function of TC spike count during trials with low base activity for the simulations. *Bottom*: Response scaling exponents as function of branching parameter σ. **c** Same as in b, but for trials with high base activity prior to stimulation. *Error bars* (a – c): mean ± SD.

Supplementary Figure 13

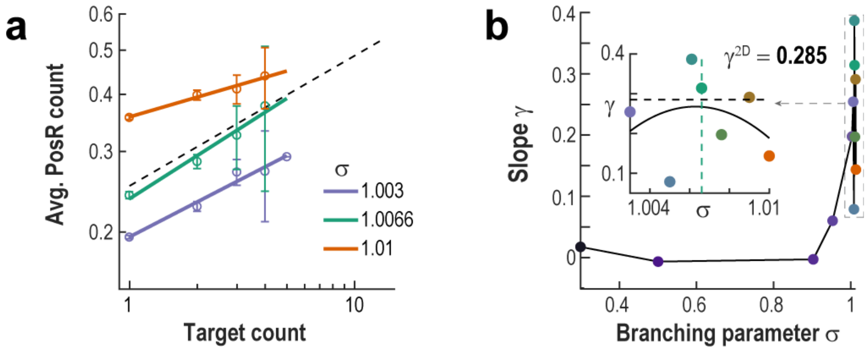

Supplementary Fig. 13 | Response of positive responders to target count during baseline for the simulations. a Mean spike count on PosR as function of spike count of TC, in log-log, during baseline, for different branching parameter σ (*colors*). Power law fits are indicated by solid lines. Dashed line indicates a slope of 0.285. *Error bars*: mean ± SD. **b** Power law exponents for the response curves of PosR for varying branching parameter, during baseline. Solid line in inset provides inverted parabola fit demonstrating steepest slope near critical dynamics.

Supplementary Figure 14

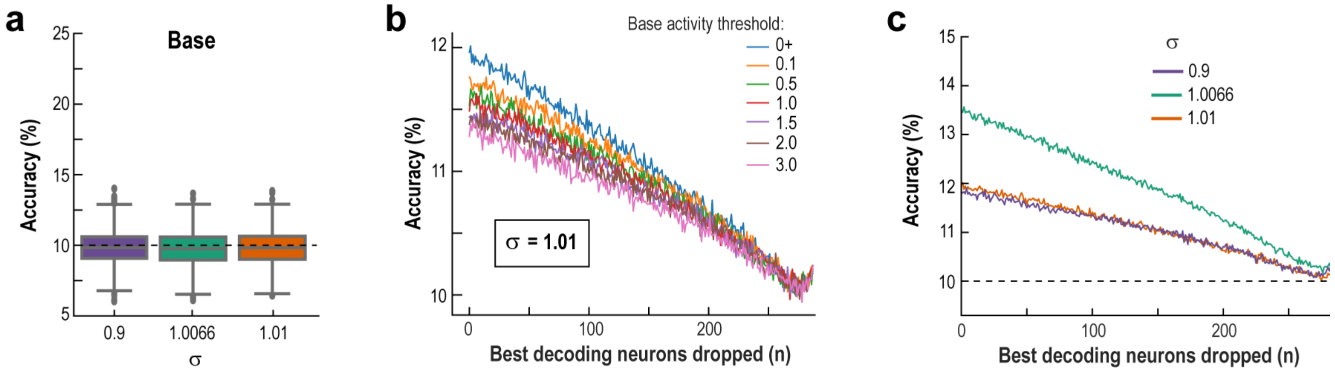

Supplementary Fig. 14 | Decoding the identity of the active neuron during baseline and controls for slightly sub-critical, critical, and slightly supercritical conditions (simulations). a F1 scores for a subcritical (*purple*), critical (*green*) and supercritical (*orange*) network, during baseline. Note that in all cases decoding is not better than chance. *Box plots*: *whiskers* represent min, median & max, while *box edges* represent 25th and 75th quartiles. *Points* represent outliers. Calculated over *n* = 100 samples for each condition. **b** TC decoding accuracy as function of best decoding neurons removed for the supercritical network (σ = 1.01) for different threshold values (*colors*; threshold indicates the minimum average activity during baseline for that trial to be included in the analysis). Trials are selected based on the average activity preceding stimulation, with the lowest threshold shown (0+) removing only trials without any spikes during baseline (~30% for this simulation). **c** Novel spikes are best decoded at criticality even when matched for trial size. Comparing decoding accuracy for different branching parameters under the same trial selection (removal of trials with no spikes during baseline: ~900 for supercritical, ~30 for critical and 0 for subcritical) and sample size matched (randomly removing trials of critical and subcritical to match supercritical condition).

Supplementary Figure 15

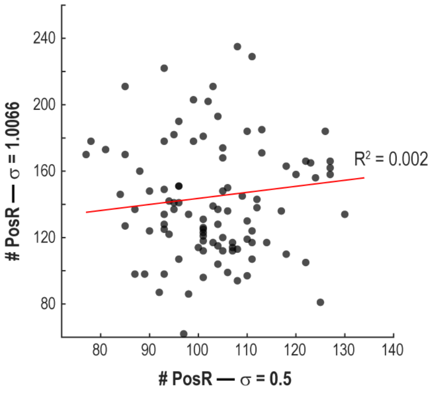

Supplementary Fig. 15 | No correlation between number of PosR for TCs between sub- and critical networks with identical connection structure. Number of PosR for each TC (*circles*) at the critical network (σ = 1.0066) as function of the same measure from a subcritical network (σ = 0.5). From the *t*-statistics of the two-sided hypothesis test we obtained p = 0.27 for the slope of the linear regression (*red line*), with R^2 = 0.002.

Supplementary Tables

Supplementary Table 1

		Experimental response scaling exponents (± 95% C.I.)		
**Condition**	**Figure**	**PosR**	**NonR**	**NegR**
Base (neuron)	S6a *left*	0.46 ± 0.07	0.52 ± 0.27	0.44 ± 0.02
Base (network)	2d, S6a *right*	0.55 ± 0.22	0.41 ± 0.27	0.76 ± 0.24
Resp (neuron)	2e, S6b *left*	0.26 ± 0.09	0.01 ± 0.10	-0.30 ± 0.18
Resp (network)	2f, S6b *right*	0.28 ± 0.10	0.04 ± 0.09	-0.07 ± 0.26
Calcium	S6c	0.18 ± 0.04	0.05 ± 0.04	-0.18 ± 0.10
Low	S12a *left*	0.29 ± 0.14	0.06 ± 0.07	-0.33 ± 0.50
High	S12a *right*	0.22 ± 0.21	-0.04 ± 0.18	-0.31 ± 0.66
No DeepIP	S8a	0.35 ± 0.18	0.08 ± 0.09	-0.78 ± 1.48

Supplementary Table 1 | Response scaling exponents for the experiments. Best fit ± 95% confidence interval shown for each condition/neuronal group.

Supplementary Table 2

Simulation response scaling exponents (± 95% C.I.)			
**Branching parameter**	**Response (Fig. 7c, d)**	**Low (Fig. S12b)**	**High (Fig. S12c)**
0.3	0.015 ± 0.048	N/A	N/A
0.5	0.045 ± 0.031	N/A	N/A
0.9	0.105 ± 0.038	N/A	N/A
0.95	0.133 ± 0.040	N/A	N/A
1	0.176 ± 0.112	N/A	N/A
1.003	0.179 ± 0.087	0.144 ± 0.150	0.124 ± 0.015
1.005	0.166 ± 0.044	0.182 ± 0.127	0.112 ± 0.043
1.0061	0.174 ± 0.032	0.201 ± 0.115	0.116 ± 0.019
1.0066	0.247 ± 0.086	0.275 ± 0.080	0.129 ± 0.039
1.0076	0.186 ± 0.082	0.469 ± 0.111	0.109 ± 0.028
1.009	0.198 ± 0.070	0.672 ± 0.108	0.112 ± 0.038
1.01	0.189 ± 0.072	0.920 ± 0.098	0.091 ± 0.013

Supplementary Table 2 | Response scaling exponents for the simulations. Best fit ± 95% confidence interval shown for each branching parameter/condition.
